# The emerging role of exosomes in mental disorders

**DOI:** 10.1038/s41398-019-0459-9

**Published:** 2019-03-28

**Authors:** Saumeh Saeedi, Sonia Israel, Corina Nagy, Gustavo Turecki

**Affiliations:** 1 McGill Group for Suicide Studies, Douglas Mental Health University Institute, McGill University, Montreal, QC Canada; 2 Department of Human Genetics, McGill University, Montreal, QC Canada; 3 Department of Psychiatry, McGill University, Montreal, QC Canada

## Abstract

Exosomes are a class of extracellular vesicles of endocytic origin, which are released by cells and are accessible in biofluids, such as saliva, urine, and plasma. These vesicles are enriched with small RNA, and they play a role in many physiological processes. In the brain, they are involved in processes including synaptic plasticity, neuronal stress response, cell-to-cell communication and neurogenesis. While exosomes have been implicated previously in cancer and neurodegenerative diseases, research regarding their role in mental disorders remains scarce. Given their functional significance in the brain, investigation in this field is warranted. Additionally, because exosomes can cross the blood–brain barrier, they may serve as accessible biomarkers of neural dysfunction. Studying exosomes may provide information towards diagnosis and therapeutic intervention, and specifically those derived from the brain may provide a mechanistic view of the disease phenotype. This review will discuss the roles of exosomes in the brain, and relate novel findings to current insights into mental disorders.

## Introduction

There has been growing interest in the development of personalized approaches in psychiatry over the last decade. Part of this drive is based on the fact that mental disorders are etiologically heterogeneous, and treatments, while effective, are helpful only in a portion of patients^1^. Additionally, patient treatment response is difficult to predict. As a result, there has been much interest in the discovery of biomarkers which, if successful, could assist clinicians in the determination of personalized treatment strategies. Biomarker research is largely based on the investigation of peripheral tissues, particularly when focused on the study of molecular markers. The relationship of peripheral findings to events taking place in the central nervous system (CNS) is an important limitation of these studies. Thus, much enthusiasm has been generated by advances in exosome research. These small extracellular vesicles are released by cells, carry molecular signals, and are involved in cellular communication^2,3^. Additionally, they can cross the blood–brain barrier (BBB), and can be detected peripherally, making them intriguing candidates in mental health biomarker discovery^2,4^.

**Fig. 1:**
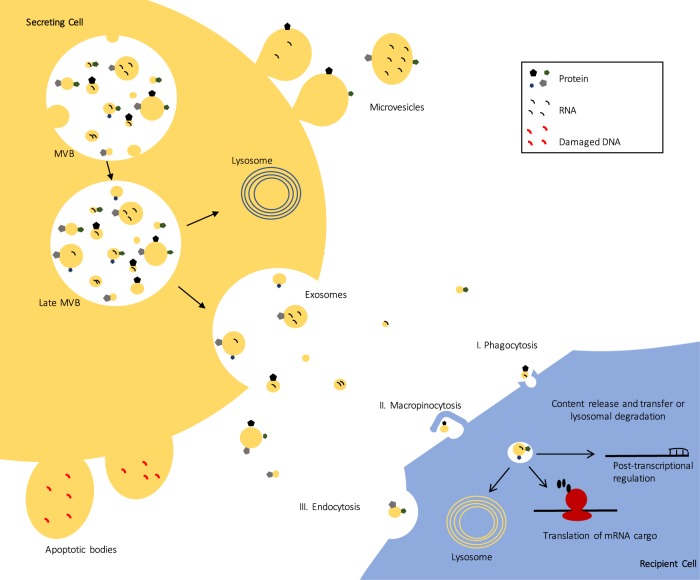
Extracellular vesicle (EV) biogenesis and cell-to-cell communication. Apoptotic bodies, the largest of the EVs, “bleb” off the cell membrane and contain material from cells undergoing apoptosis to signal to macrophages. Microvesicles bud off the plasma membrane and contain cargo that can facilitate signaling to recipient cells. Exosomes are the smallest of the vesicles, and are first made as a population of heterogeneous intraluminal vesicles in the multivesicular body (MVB). The MVB has two fates, either fusing with the lysosome, or fusing with the plasma membrane where they are released as exosomes. Exosomes can be taken up by other cells either by endocytosis, micropinocytosis, or phagocytosis where its contents can effectively influence cellular processes. Contents can be involved in transcriptional regulation, or mRNA cargo can be transcribed in recipient cells

The term “extracellular vesicles” (EVs) encompasses a group of cell-derived vesicles produced by most, if not all cell types, that are released to the extracellular environment^3^. Growing evidence suggests that these vesicles have a functional impact on physiological processes, and are especially vital in cell-to-cell communication^2,3^. Since the EV field has grown, different types of vesicles have been described, which differ in their properties, as well as their biogenesis (Fig. 1)^3^. The three main types are: apoptotic bodies (500–2000 nm), microvesicles (50–1000 nm), and exosomes (40–200 nm)^5^. Apoptotic bodies are EVs that bud off the membrane of cells undergoing apoptosis, and are typically engulfed by macrophages^3^. Microvesicles directly bud off the plasma membrane and contain a range of cargo that is delivered to neighbouring cells^3^. Exosomes, which are the smallest class of extracellular vesicles, first develop as intraluminal vesicles (ILV) through the inward budding of the multivesicular body (MVB)^3,6^. The MVB has two potential fates; it can either fuse with the lysosome, leading to the degradation of its contents, or fuse with the plasma membrane, and release its ILV contents as exosomes into the extracellular space (Fig. 1)^3^. In terms of cargo, exosomes contain a variety of biological materials including proteins, lipids, and nucleic acids^3^. Notably, compared to plasma, saliva, or other biological fluids, exosomes are highly enriched in microRNA (miRNA)^5,7^. The majority of miRNA that can be accessed from serum or saliva are contained in exosomes, and some miRNA appear to be dependent on exosomes as they go undetected as free floating molecules in biofluids^7^. Although there is currently no concrete evidence to show there is a miRNA sorting mechanism for exosomes, there is evidence to suggest that this is a possibility. MiRNA profiles of exosomes do not always match the profiles of parent cells, and observations of miRNA enrichment in exosomes further suggests a mechanism of selective miRNA export^8,9^. Additionally, miRNA expression in exosomes can be altered based on physiological changes such as disease state, making the miRNA cargo intriguing candidates for investigation. To date, there is evidence of altered exosomal cargo in disease development and progression in pathologies such as cancer^10^ and neurodegenerative diseases^11,12^. However, to date there is only one study that has investigated exosomal miRNA cargo alterations in mental disorders^13^. Banigan et al.^13^ used exosomes from frozen postmortem prefrontal cortex to study miRNA alterations in schizophrenia and bipolar disorder^13^. They found that miR-497 in schizophrenia patients and miR-29c in bipolar patients to be upregulated compared to controls^13^. This early work opens up interesting possibilities for the study of exosomes in mental disorders, demonstrating that miRNA cargo may be interesting to investigate in these phenotypes. Indeed, miRNAs have already been implicated in several mental disorders, such as depression, schizophrenia, anxiety, and bipolar disorder, including being implicated as candidate peripheral biomarkers for disease development and treatment response^14–17^.

In recent years, efforts to characterize exosomal release and uptake have had important implication for their role in the CNS. Previous studies have demonstrated that exosomes and their cargo play a role in normal communication in the CNS, as well as nerve regeneration, synaptic function, plasticity, and immune response^18–20^. In addition to their critical role in normal brain function, exosomes have also been implicated in the propagation of neurodegenerative diseases^12,21^. Given exosomes’ role in normal brain physiology, and their contribution to other CNS disease states such Parkinson’s^12^, and Alzheimer’s^21^ it is reasonable to hypothesize exosomes may play a significant role in the pathogenesis of mental disorders. Exosomes have been found to play a role in processes that have long been hypothesized to be involved in psychopathology of mental disorders, such as neuroinflammation^22^, neurogenesis^23^, plasticity^24,25^, and epigenetic regulation^26^. Additionally, their ability to cross the blood–brain barrier (BBB) suggests that exosome content in the CSF and plasma may reflect ongoing neural processes^27^. Therefore, information from neural-derived exosomes found in peripheral sources might be able to provide relatively non-invasive markers of clinical utility for mental disorders.

This review highlights the role of exosomes in the CNS. Particularly, it focuses on their role and cargo in the brain; their ability to cross the blood–brain barrier; and their release and transfer. Recent insights in the properties of exosome signaling in the brain are then related to existing pathophysiological perspectives of mental disorders. Results discussed here support the notion that the function of exosomes in the brain may align with neurobiological theories of mental disorders, and that exosomes have the potential to be strong biomarker candidates for this psychopathology.

### Cell communication via exosomes in the brain

**Fig. 2:**
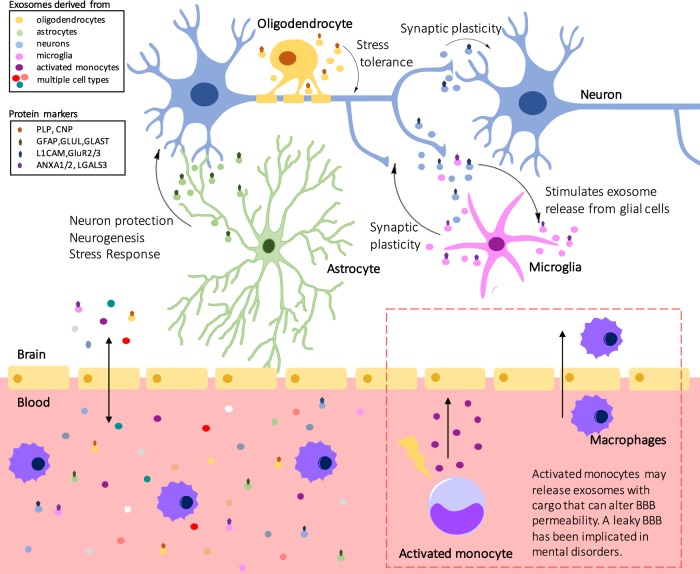
The role of exosomes in the brain. Exosome signalling is involved in many physiological brain processes. Changes in many of these processes have been previously associated in mental disorders. For example, activated monocytes release exosomes that can influence BBB permeability. A leaky BBB is associated with neuroinflammation, and has been previously implicated in schizophrenia, bipolar disorder, and major depressive disorder. Additionally, exosomes carry markers from parent cells that may help them be distinguishable in biofluids

Exosomes play important roles in cell communication in the CNS, acting on both neighbouring and distal cells (Fig. 2)^28^. These vesicles can act as important vehicles of communication both within a cell type, and between different cell types. Evidence from multiple studies demonstrates that exosome release from cells in the CNS is a highly regulated process, with release regulated by synaptic glutamatergic activity and calcium influx^18,29^. Neuronal exosome release is triggered by Ca^2+^ entry through N-methyl-D-aspartate (NMDA) and α-amino-3-hydroxy-5-methyl-4-isoxazolepropionic acid (AMPA) receptors at glutamatergic synapses, suggesting that exosome release may be part of normal synaptic physiology^18^. Additionally, the controlled subcellular location of release of exosome has been documented in neurons; however, the mechanism is unclear^30^. MVBs are ~50 times more represented in soma or dendrite compartments compared to axons^30^. Although the mechanism for preferred compartmentalization is unknown, these areas of specific enrichment further support both, a role for exosomes at synapses, and their highly regulated release.

Serotonin (5-HT) has also been implicated in the release of exosomes from non-neuronal cells types in the brain. Serotonin can increase cytosolic levels of calcium, which in turn, stimulates the release of exosomes from primary microglial cells^29^. Dysregulation in serotonin pathways has been implicated in depression, anxiety, bipolar disorder, and schizophrenia, with its receptors being targets of some of the most commonly prescribed drugs^31–34^. Given that microglial release of exosomes can be regulated by serotonin, and serotonin is often found to be altered in mental disorders, it follows that microglial exosome release may also be modified in these disorders. Both neurotransmitter release and cell communication are important factors in psychopathology^35^. It will therefore be important to understand the role exosomes may play in the etiopathogenesis of mental disorders given their prominence in the regulation of cell communication, and their regulation via neurotransmitters.

Basic neuron-to-neuron communication can occur through exosome release and uptake^36^. Strikingly, it was demonstrated that a subpopulation of neuron-internalized exosomes can be re-secreted along with the recipient neuron’s endogenous exosomes, seemingly to facilitate long-distance interactions^36^. While the eventual fate of these exosomes remains undetermined, these findings demonstrate the ability of exosomes to mediate communication within a cell type and the potential for widespread signaling^36^. Additionally, neuron-to-neuron signalling via exosomes has found to be involved in important processes including synaptic plasticity (Fig. 2)^37^.

Exosomes are also mediators of cellular communication between cell types, with evidence of glial-to-neuron communication^38^. In a feedback loop-like manner, neurotransmitter release can stimulate oligodendrocyte exosome secretion, while neurons are able to internalize oligodendrocyte exosomes and utilize their cargo^39^. The internalization of oligodendrocyte-derived exosomes by neurons can result in greater tolerance to stress and increased viability resulting in a form of cellular protection (Fig. 2)^39^. Additionally, neuron to microglial communication also occurs via exosomes (Fig. 2). When neurons were co-cultured with microglial cells, neuron-derived exosomes were internalized by the microglial cells^19^. This internalization resulted in an enhancement of the cells removing degenerative neurites^19^.

Although knowledge regarding astrocyte-neuron communication via exosomes remains scarce, there is evidence to suggest that it does occur, and this method of communication is critical for neuronal cell survival (Fig. 2). Prion protein (PrP) is an important protective protein against oxidative stress. Protection of neurons via astrocyte-derived exosomes was dependent on astrocyte-derived exosomal PrP transportation into neurons^40^. Considering all of these reports together, cell communication via exosomes is emerging as an important regulator of neuron protection and synaptic plasticity (Fig. 2), and their dysregulation has been implicated in the pathophysiology of mental disorders, such as bipolar disorder, major depressive disorder, and schizophrenia^41–44^. Neuroprotective signaling is required for proper growth and survival of neurons, and alterations in the number neurons, glia, and neuropils have previously been reported in mental disorders^42,43^.

After release into the extracellular space, exosomes can be internalized by recipient cells via several mechanisms including phagocytosis, micropinocytosis, endocytosis, and plasma membrane fusion (Fig. 1.)^45^. Most investigations of exosomes in the CNS report endocytosis-based uptake^46,47^ but, there is also evidence to suggest that uptake depends on the recipient cell type. Specifically, it was found that exosomes released from neuroblastoma cells are preferentially endocytosed by glial cells, whereas exosomes released from cortical neurons are selectively endocytosed by other neurons^48^.

Once exosomes have been internalized by recipient cells, their exosome cargo may elicit an effect on the cell (Fig. 1)^9^. One of the first studies to demonstrate functional exosome cargo transfer to recipient cells was done by Valadi and colleagues in 2007^9^. After incubation and transfer of mouse exosomes to human cells, three new mouse proteins were found in recipient human cells, therefore providing evidence that exosome mRNA can be translated in recipient cells^9^.

Since the original discovery that exosome cargo transfer displays functional effects in the recipient cell, there have been several studies investigating this mechanism as a means of cellular communication in both disease and healthy states. For example, an exosome’s ability to spread cargo and elicit an effect on recipient cells has been identified as a potential pathway involved in cancer development and progression. Exosomes isolated from colon cancer cells expressing a mutant form of the protein K-RAS (KRAS) contain the mutant KRAS along with numerous proteins that have the ability to promote tumor progression^49^. These exosomes, which can be internalized by wild-type colon cells, can transfer the mutant protein to healthy cells, effectively leading to enhanced growth of these cells^49^. In the brain, communication via exosomes has been found to play a role in Alzheimer’s disease progression by neuron-to neuron transport of misfolded amyloid-beta oligomers^50^. Using an in vitro model, exosome formation and secretion was blocked via the siRNA knockdown of proteins required for these functions, and the spread of the oligomers was in turn also blocked^50^. Although there are clear phenotypic and mechanistic differences between cancer, Alzheimer’s, and mental disorders, and given the continuum in which mental disorders lie, we would expect quantitative and not dichotomous changes in this phenotype. Nonetheless, results from the studies above show that exosomes can propagate disease spreading through cargo transfer. Since miRNAs have previously been implicated mental disorders^14–17^, it is possible that exosome miRNA transfer is contributing to the progression of phenotype, as well as individual symptoms. It would be of interest to investigate whether miRNA associated with psychiatric phenotypes are packed into exosomes, and whether these exosomal miRNA profiles are altered in mental disorders.

### The ability of exosomes to cross the blood–brain barrier (BBB)

The BBB lies at the interface of the peripheral circulatory system and the CNS, acting as a highly selective membrane that protects the brain’s microenvironment and preserves homeostasis^51^. The BBB is mainly comprised of brain macrovascular endothelial cells (BMECs) and tight junctions to prevent the transfer of potentially toxic compounds between the blood and the brain^52^. Besides transmembrane diffusion of small (<400 Da) lipid soluble molecules, the BBB allows for selective transport of some compounds into and out of the brain^52^. Transport of material across the BBB can be either transcellular through BMECs, or paracellular through junctions between BMECs^53^.

The findings that exosomes can cross the BBB, and that its contents remain active, have been instrumental in biomarker research with exosomes and their use as a drug delivery system. Alvarez-Erviti et al. demonstrated effective delivery of siRNA to the brain via systemic injection of exosomes in mice^26^. They engineered dendritic cells to express lysosome-associated membrane protein 2 (Lamp2b), an exosomal membrane protein^26^. By fusing Lamp2b to a rabies virus glycoprotein (RVG) peptide that is specific to the CNS, the exosomes were targeted exclusively to the brain^26^. These exosomes delivered GAPDH siRNA, which resulted in specific gene knockdown exclusively in the brain^26^. Later studies have been successful in delivering exosomes via intranasal injection in mice to the brain^54^. Most recently, a study using rats identified that a fluorescently tagged protein expressed selectively in brain tissue could be recovered in small EVs (those with the same characteristics as exosomes) in their blood^4^. This study provides evidence of communication via exosomes from the brain to the rest of the body^4^. Evidence from these studies support the notion that exosomes cross the BBB in a bi-directional manner; however, their exact method of crossing remains unclear.

Much of the current research examining how exosomes can cross the BBB points to the transcellular method of transport through BMECs via the different mechanisms of endocytosis. The transfer of EVs derived from human erythrocytes in an in vitro BBB model was dependent on the adsorptive-mediated transcytosis method of transport^55^. Although the EVs did cross under healthy and inflammatory conditions, EVs movement across the BBB was significantly higher after the peripheral administration of lipopolysaccharides^55^. Another study by Chen et al.^53^ demonstrated exosomes crossing a BBB model using transcellular BMEC endocytosis in healthy and stroke-like condition, suggesting that exosomes retain their ability to cross during stressful states^53^. This group demonstrated that exosomes are internalized through endocytosis, and accumulate in endosomes. After MVB formation, the exosomes are then released on the other side of the BMEC monolayer. The data suggest that exosomes derived from human embryonic kidney cells could cross using multiple pathways of endocytosis^53^. Using an inhibitor for clathrin-dependent endocytosis, chlorpromazine (CPZ), which transfers clathrin from the surface of cells to intracellular endosomes^56^, there was a decrease in exosome transcellular migration^53^. This suggests that clathrin-dependent endocytosis may be involved in transportation of exosomes across the BBB^53^. Additionally, methyl-β-cyclodextrin (MβCD), which removes cholesterol from the plasma membrane^56^, and filipin III, which binds to cholesterol^56^, also significantly reduced exosomes crossing the BBB^53^. This result suggests that caveolae-dependent endocytosis is another possible route of migration. It is rather likely that uptake of exosomes in BMEC will depend on specific ligand receptors or lipid rafts, and mechanisms of exosome uptake may depend on the cell of origin. Exosomes from different cells may have different cargo including protein and lipids, potentially altering their method of crossing the BBB^57^. Furthermore, disease state may impact the method used to cross the BBB, as cargo can change upon disease state^13,57^.

In addition to crossing, recent studies have elucidated a role for exosomes in increased permeability of vascular barriers of the BBB. For example, exosomes secreted from breast cancer cells uniquely express miR-105, which directly targets the tight junction protein ZO-1^58^. This exosome transfer of miR-105 destroys tight junctions and the integrity of the BBB^58^. In addition, claudin-5 (Cldn5) has been found to be encapsulated in exosomes, which is a tight junction protein present in the BBB^59^. When Cldn5 is knocked out in mice, it results in the loosening of the BBB^60^, suggesting that exosomes carrying Cldn5 may play a role in BBB integrity. Interestingly, a decrease in Cldn5 is sufficient to induce depressive-like behaviors in these mice, and treatment with antidepressants increases Cldn5 levels and promotes disease resilience^61^. A leaky BBB is associated with neuroinflammation—a prominent theory of mental disorders^62,63^. Therefore, the possibility of exosomes influencing the integrity of the BBB may also suggest a role for exosomes in neuroinflammation and the pathogenesis of mental disorders^63^. Taking it one step further, a leaky BBB state in mental disorders may be initiated by exosomes released from cells being influenced by this disease state.

Crossing the BBB allows for communication between the periphery and the CNS, therefore communication via exosomes may account for some of the systemic changes observed in several mental disorders. For example, the bi-directional communication between the gut microbiome and the brain has previously been associated with mental disorders, with most attention focusing on its link to depression^64,65^. Additionally, dysregulation of systemic immune response has been well documented in mental disorders including depression, schizophrenia, and bipolar disorder^66^. It is possible that cells responding to a psychiatric state may release exosomes that, in turn, affect the peripheral inflammatory response or the gut microbiome. In addition to exosomes being a link between the CNS and periphery in mental disorders, peripheral access to CNS-derived vesicles make them ideal carriers of potential biomarkers. These vesicles are better suited to providing insight into changing mechanisms in the CNS of affected individuals.

Since the discovery of exosomes’ ability to cross the BBB, there has been increasing interest in their ability to act as a drug delivery system to the brain. They have been found to be a promising vehicle for drug delivery in many types of cancers, both in vivo and in vitro^67^ showing they are able to deliver drugs across the BBB. In cancer, reports have shown that delivery of drugs across the BBB resulted in decreased markers for brain tumor growth^68^. Other than cancer, exosomes have been found to be an effective drug delivery system for brain-related diseases. A formulation of catalase, a promising treatment for Parkinson’s disease, can be loaded into exosomes and reach target neurons where the drug then accumulates^69^. In a study by Liu et al., exosomes expressing neuron-specific rabies viral glycoprotein (RVG) peptide were used to deliver opioid receptor mu (MOR) siRNA into the brain to treat morphine addiction^70^. The exosomes efficiently delivered the MOR siRNA into the mouse brain and reduced MOR, resulting in the inhibition of morphine relapse^70^. Their role as a drug delivery system seems extremely promising, and this could be an interesting line of research for further investigation, as there are many advantages to implementing targeted treatment in mental disorders. Using nanotechnology for drug delivery to the brain has the potential to alleviate some of the peripheral symptoms in mental disorders, as well as solve the problem of delivery across the BBB and drug solubility^71^.

### Exosome biogenesis in disease states

Exosomes were once thought to be a rather homogenous population of vesicles; however, more recently, studies have found that they are rather diverse^72^. Exosome biogenesis appears to be a more dynamic process, with heterogeneous populations of exosomes being produced. A study by Willms et al.^72^ identified a large (75–200 nm in size) and small (most <100 nm) population of exosomes from the same cell type^72^. They repeated this experiment with different cell types, as well as plasma, and found similar results^72^. Results suggested that the two different populations had distinct protein and RNA profiles^72^. In the smaller exosomes they identified less individual proteins (110 proteins compared to 254 in larger vesicles), suggesting the smaller vesicles had more specific types of protein cargo^72^. Additionally, the smaller vesicles were enriched in smaller RNA molecules compared to the larger vesicles^72^. Although currently there is no evidence for roles of the different sized exosomes, it would not be surprising if smaller exosomes contained less, or smaller material as briefly eluded above^72^. This study used nanoparticle tracking analysis (NTA) to characterize exosomes by size; however, there are multiple technologies that can be used for this measurement. Particle size profiling and/or quantification can also be measured using technologies including tunable resistive pulse sensing^73^, high resolution flow cytometry^74^, and optical disc technology^75^. Consistencies in technologies is imperative as each technology may yield different results from the same sample^76^.

Although there is not much evidence in changes in size given a disease state, there is evidence to suggest that biogenesis is affected in disease states as exosome quantity may be altered. Because the field of exosomes in mental disorders is in its infancy, changes in exosome biogenesis have yet to be studied thoroughly. Exosome biogenesis seems to be enhanced in cancer, with tumor cells secrete more exosomes than non-tumor cells, and exosome levels of cancer patients are often elevated^77^. In one specific investigation, quantification of exosomes from plasma showed that esophageal cancer patients expressed higher exosome levels compared to patients with a non-malignant tumour^78^. Another study used plasma from patients with ovarian cancer and found similar results^79^. Subjects with malignant tumours had more exosomes than those with benign^79^. And subjects in the malignant and benign groups had more exosomes than healthy controls^79^. Escalating amounts of exosomes may result in an increase in signalling between cells. Additionally, altered cargo in these vesicles may aid in tumor and disease progression.

Although most of the research on changes in exosome biogenesis has been conducted in cancer, this is some evidence to show that these changes may occur in other disease states affecting the brain. Enriched exosome secretion is documented in brains of individuals with Down syndrome, and a knockdown of exosome secretion resulted in worsening endosomal pathology in fibroblasts from these patients^80^. Additionally, an increase in EV-associated protein, suggesting an increase in EVs, was observed in serum from subjects with autism spectrum disorder (ASD)^81^. Results from a study in 2017 showed that individuals with HIV had less exosomes in plasma than healthy controls^82^. Neurological deficits, HIV-associated neurocognitive disorder (HANDS), develops in a portion of adults with HIV^82^. Patients that were neuropsychologically impaired had fewer neuron-derived exosomes than patients who were neuropsychologically normal^82^. Fewer exosomes may result in a change or lack of signaling between cells. Results from the above studies suggest that exosomes are extremely heterogeneous in nature and that biogenesis can be altered in disease states. Investigation into exosome biogenesis may provide more insight into the etiology of mental disorders. Identifications of altered amounts or sizes in mental disorders may provide more insight into changes in cellular communication occurring within the disease state.

### Possible role of exosomes in the pathogenesis of mental disorders

Current evidence for exosome signaling in the brain points toward their role in transcriptional regulation^83^, neurogenesis^23^, plasticity^24,25^, and neuroinflammation^22,62^. Changes in these mechanisms have also been previously implicated in mental disorders, providing reason to hypothesize that exosomes may be involved in these phenotypes.

Neurogenesis has been previously implicated in schizophrenia and depression, and research suggests that these disorders are associated with impaired adult hippocampal neurogenesis (AHN)^23^. Protein analysis of exosomes in the CNS reveals cargo involved in modulating adult neurogenesis^23^. Furthermore, the injection of cultured exosomes containing known pathogens into the dentate gyrus is sufficient to impair AHN in mice^84^. CSF-derived factors and substances such as corticosteroids and cytokines may trigger the release of astrocytic exosomes containing several miRNAs important for neurogenesis, stress response, and cell survival^23^. Thus, it is possible that exosomes are involved in both the maintenance and hindrance of adult neurogenesis.

Protein analyses of exosomes in the CNS reveal that some cargo is involved in modulating synaptic plasticity, suggesting exosomes may play a role in this process^24,25^. For example, microtubule-associated protein 1B (MAP1b), a protein associated with synaptic plasticity, was identified in exosomes from depolarized human neurons in culture^85^. When microglial cells were incubated with neuron-derived exosomes, removal of neurites was accelerated by increasing the expression of complement component 3 (C3) in the microglial cells^19^. Neuron-to-glial signalling via exosomes is one mechanism where active synapses stimulate the pruning of those that are inactive, thereby promoting synaptic plasticity^19^.

There is also mounting evidence for the role of exosomes in neuroinflammation. Upon exposure to the pro-inflammatory cytokine tumour necrosis factor (TNF), exosomal protein cargo from brain endothelial cells is altered^86^. These exosomes contained proteins involved in TNF and NF-ĸB signaling pathways^86^. The neuroinflammation caused by TNF relates to the low-level, chronic neuroinflammation associated with certain forms of psychopathology, particularly depression^62^. Additionally, monocytes that are activated by interferon alpha and/or lipopolysaccharides release exosomes that carry altered miRNA profiles^22^. These exosomes can alter BMECs and initiate an inflammatory response^22^. Together, with studies on BBB permeability in mental disorders, the evidence above demonstrates that alterations in BMEC could result in a leaky BBB, leading to an increase in neuroinflammation and onset, or progression of disorders (Fig. 2). Exosome involvement in neuroinflamation has also been documented in mental disorders. EVs isolated from patient serum with ASD stimulated cultured human microglial cells to secrete more pro-inflammatory cytokine interleukin IL-1β^81^. Another study used an ELISA based method to detect inflammatory markers, in what is suggested to be neural-derived exosomes in a plasma sample. Anti-SNAP25, a neuron marker, was used as the capture antibody, and anti-CD81, a known exosome marker, along with inflammatory markers were used as the detection antibody^87^. After normalization, the ratio of IL34/CD81 was significantly higher in patients with major depressive disorder (MDD) compared to controls, suggesting increased inflammation^87^. However, it is important to note that although CD81 is a known exosome marker, it is not exclusive to exosomes and the ELISA based method may be detecting non-EV bound proteins.

Interestingly, central inflammation can be detected systemically via EVs, making them ideal candidates for biomarkers of mental disorders. In one recent study by Couch et al. (2017), brain injury was shown to increase EV release in rats^88^. EVs from those rats were collected, and injected into healthy rats. The EVs were taken up by the liver where they initiated a systemic acute phase response (APR), a reaction to inflammation for the activation of an early-defense system^88^. Alterations to the periphery have also been found to affect CNS function as demonstrated by injecting (via tail-vein injection) peripherally-derived exosomes from immune-challenged mice. This led to increased CNS expression of pro-inflammatory cytokine mRNA and associated miRNA in recipient mice^20^. Given that exosomes can elicit a peripheral response to inflammation^88^, it would be interesting to investigate whether exosomes may partially explain peripheral changes, such as changes within the gastrointestinal system and gut microbiome observed in mental disorders^65^.

There is also evidence that exosomes may be a transfer vehicle for translational regulators, specifically via the transfer of miRNA cargo^9,83,89^. Once exosomes fuse with target cells, they may transfer their miRNA content to recipient cells, where they remain functional^9^. Sustained changes in gene expression, through epigenetic modifications, are associated with mental disorders^14–17^. Expression levels of numerous miRNAs are demonstrably altered in serum^17,90^ and in postmortem brain tissue of psychiatric patients^91,92^. The EV cargo, specifically miRNA, could potentially explain in part the modifications in gene expression observed in mental disorders.

Taken together, exosome signalling appears to play a role in gene regulation, plasticity, neurogenesis, and neuroinflammation. Should exosomes mediate such mechanisms in the brain, these nano-vesicles might be critical to further understanding neurobiological changes occurring in mental disorders.

### Biomarker potential of exosomes

The ability of exosomes to readily cross the BBB is an important property that renders them as particularly good biomarkers for CNS diseases and treatment response. Of particular interest is the ability to characterize exosomes based on their cell of origin, potentially providing an extra layer of insight into the disease of interest. Currently, much of the cell-specific exosome research is performed in cell culture; however, exosomes from different cell types are diverse, and there has been a recent surge in interest for identifying exosomes of a specific origin from biological fluids^93^. The potential to access centrally-derived material in the periphery may provide compelling information about the mechanism of a disease, by way of a clinically accessible biomarker.

A mixed population of exosomes (from multiple cell types) can be isolated from biofluids using multiple methods including ultracentrifugation, immunomagnetic beads, and chromatography^94,95^. Additionally, exosomes have a lipid bilayer; therefore RNAse treatment prior to use will ensure that cargo used downstream was encapsulated within the vesicle^96^. This mixed population of exosomes may be identified using western blots or mass spectrometry using proteins which are involved in biogenesis of ILVs, including tetraspanins and proteins involved in the ESCRT machinery needed for biogenesis^97^. It is important to note that many of these markers are not exclusive to exosomes, and further characterizations of exosomes is required.

It is possible to take this one step further and enrich for exosomes derived from a specific cell type from the mixed population of exosomes using cell-specific markers, as exosomes have been found to carry proteins specific to their cell of origin. In psychiatry, investigating cells from the CNS may provide insights toward mechanisms of disease in the brain. Isolating exosomes from those cells that have been implicated in mental disorders may bridge the gap between peripheral biomarkers and mechanistic insight to the disease.

Exosomes released from developing and mature hippocampal neurons contain L1 cell adhesion molecule (L1CAM), and the GluR2/3 subunits of glutamate receptors, both of which are known neuronal markers^18,98^. Protein markers, such as glial fibrillary acidic protein (GFAP), glutamine aspartate transporter (GLAST), and glutamine synthetase (GLUL), can be used to enrich for astrocytic-derived exosomes^11^. Additionally, myelin proteolipid protein (PLP) and 2’, 3’-cyclic nucleotide 3’-phosphodiesterase (CNP) have been identified on exosomes derived from oligodendrocytes^99^. Enriching for a specific cell-derived population of exosomes allows for examination of target cells of interest. In the biomarker field, this may allow for greater connections to form between the marker and mechanisms of disease.

In the last few years, research has been conducted with neuron-derived exosomes to try and answer questions of brain-related disorders from blood biopsies. Sun et al.^82^ use exosomes isolated from plasma to enrich for neuron-derived exosomes. In doing so, the group identified that both the number of neural-derived exosomes as well as levels of High-mobility group box 1, Neurofilament light, and Amyloid β-proteins may act as potential biomarkers of neuropsychological impairment in HIV^82^. Neuronal-derived EVs were isolated and concentrations of tau, Aβ42, and IL-10 were elevated in military personal with mild traumatic brain injuries compared to controls^100^. Neural-derived exosomes from plasma have also been used in a pilot study to investigate protein biomarkers for patients with MDD^87^. Additionally, other cell-derived exosomes have been studied in the context of other brain-related disorders. Cargo proteins from astrocytic-derived exosomes have been studied for mechanistic insight into Alzheimer’s disease^11^. The ability to access neural-derived exosomes in plasma shows promising clinical utility in psychiatry.

### Future directions

Although the field of exosome investigation remains relatively novel, compelling evidence from other domains indicates that studying exosomes can provide insight into disease mechanisms and processes associated with mental disorders and treatment response. Currently, much of the research on exosomes fixates on biomarkers of disease state, and their ability to mediate cell-to-cell communication. However, more work is needed with respects to mechanisms of bi-directional transfer of exosomes across the BBB. Future studies of exosomes in psychiatry should focus on profiling changes in size or number of exosomes released, and changes in cargo. Additionally, this type of work can be further extended by investigating these differences in a specific cell type. Exosomes derived from cells in the CNS have immense biomarker potential, as they may reflect physiological changes in mental disorders, which can be accessed in the periphery.
